# Inequalities of extended beta and extended hypergeometric functions

**DOI:** 10.1186/s13660-016-1276-9

**Published:** 2017-01-06

**Authors:** Saiful R. Mondal

**Affiliations:** Department of Mathematics and Statistics, College of Science, King Faisal University, Al-Hasa, Hofuf, 31982 Saudi Arabia

**Keywords:** 33B15, 33B99, extended beta functions, extended hypergeometric functions, log-convexity, Turán-type inequalities

## Abstract

We study the log-convexity of the extended beta functions. As a consequence, we establish Turán-type inequalities. The monotonicity, log-convexity, log-concavity of extended hypergeometric functions are deduced by using the inequalities on extended beta functions. The particular cases of those results also give the Turán-type inequalities for extended confluent and extended Gaussian hypergeometric functions. Some reverses of Turán-type inequalities are also derived.

## Introduction

For $\operatorname {Re}(x)>0$, $\operatorname {Re}(y)>0$, and $\operatorname {Re}(\sigma )>0$, define the functions

1 $$\begin{aligned} \mathtt{B}_{\sigma }(x,y):= \int_{0}^{1} t^{x-1} (1-t)^{y-1} \exp \biggl( - \frac{\sigma }{t(1-t)} \biggr)\,dt. \end{aligned}$$

The function $\mathtt{B}_{\sigma }$ is known as the extended beta function, which was introduced by Chaudhry *et al.* [1]. They discussed several properties of this extended beta functions and also established connection with the Macdonald, error, and Whittaker functions (also see [2]).

Later, using this extended beta function, an extended confluent hypergeometric functions (ECHFs) were defined by Chaudhry *et al.* [3]. The series representation of the extended confluent hypergeometric functions is

2 $$\begin{aligned} \Phi_{\sigma }(b ; c; x) := \sum _{n=0}^{\infty} \frac{ B_{\sigma }(b+n, c+n)}{B(b, c-b)} \frac{ x^{n}}{n!}, \end{aligned}$$

where $\sigma \geq 0$ and $\operatorname {Re}(c) > \operatorname {Re}(b) >0$. For $\sigma >0$, the series converges for all *x*, provided that $c \neq 0, -1, -2,\ldots$ .

The ECHFs also have the integral representation

3 $$\begin{aligned} \Phi_{\sigma }(b ; c; x) :=\frac{1}{B(b, c-b)} \int_{0}^{1} t^{b-1} (1-t)^{c-b-1} \exp \biggl( x t- \frac{\sigma }{t(1-t)} \biggr)\,dt. \end{aligned}$$

Similarly, the extended Gaussian hypergometric functions (EGHFs) can be defined by

4 $$\begin{aligned} F_{\sigma }(a, b ; c; x) := \sum _{n=0}^{\infty} \frac{ B_{\sigma }(b+n, c-b)}{B(b, c-b)} \frac{(a)_{n}}{n!} x^{n}, \end{aligned}$$

where $\sigma \geq 0$, $\operatorname {Re}(c) > \operatorname {Re}(b) >0$, and $|x|<1$. For $\sigma >0$, the series converges when $| x|< 1$ and $c \neq 0, -1,-2, \ldots$ .

The EGHFs also have the integral form

5 $$\begin{aligned} F_{\sigma }(a, b ; c; x) :=\frac{1}{B(b, c-b)} \int_{0}^{1} t^{b-1} (1-t)^{c-b-1} (1-x t) ^{-a} \exp \biggl( - \frac{\sigma }{t(1-t)} \biggr)\,dt. \end{aligned}$$

Note that for $p=0$, the series (2) and (4) respectively reduce to the classical confluent hypergeometric series and the Gaussian hypergeometric series.

The aim of this article is to study the log-convexity and log-convexity of the mentioned three extended functions. In particular, we give more emphasis on the Turán-type inequality [4] and its reverse form.

The work here is motivated by the resent works [5–10] in this direction and references therein. Inequalities related to beta functions and important for this study can be found in [11, 12].

In Section 2.1, we state and prove several inequalities for extended beta functions. The classical Chebyshev integral inequality and the H$\ddot{\text{o}}$lder-Rogers inequality for integrals are used to obtain the main results in this section. The results in the Section 2.1 are very useful in generating inequalities for ECHFs and EGHFs, especially, the Turán-type inequality in Section 2.2. The log-convexity and log-convexity of ECHFs and EGHFs are also given in Section 2.2.

## Results and discussion

### Inequalities for extended beta functions

In this section, applying classical integral inequalities like Chebychev’s inequality for synchronous and asynchronous mappings and the Hölder-Rogers inequality, we derive several inequalities for extended beta functions. Few inequalities are useful in the sequel to derive the Turán-type inequalities for EGHFs and ECHFs.

#### Theorem 1

*Let* $x, y, x_{1}, y_{1} > 0 $ *be such that* $(x-x_{1})(y-y_{1}) \geq 0 $. *Then*

6 $$\begin{aligned} B_{\sigma }(x, y_{1})B_{\sigma }(x_{1}, y) \leq B_{\sigma }( x_{1}, y _{1}) B_{\sigma} (x, y) \end{aligned}$$

*for all* $\sigma \geq 0$.

#### Proof

To prove the result, we need to recall the classical Chebyshev integral inequality ([13], p.40): If $f, g : [a, b] \to \mathbb{R}$ are synchronous (both increase or both decrease) integrable functions and $p: [a, b] \to \mathbb{R}$ is a positive integrable function, then

7 $$\begin{aligned} \int_{a}^{b} p(t) f(t)\,dt \int_{a}^{b} p(t) g(t)\,dt \leq \int_{a}^{b} p(t)\,dt \int_{a}^{b} p(t) f(t) g(t)\,dt. \end{aligned}$$

Inequality (7) is reversed if *f* and *g* are asynchronous.

Consider the functions $f(t):=t^{x-x_{1}}$, $g(t):=t^{y-y_{1}}$, and

$$p(t):= t^{x_{1}-1} (1-t)^{y_{1}-1} \exp \biggl( - \frac{ \sigma }{t(1-t)} \biggr). $$

Clearly, *p* is nonnegative on $[0,1]$. Since $(x-x_{1})(y-y_{1}) \geq 0 $, it follows that $f'(t)=(x-x_{1}) t^{x-x_{1}-1}$ and $g'(t)=(y-y_{1})t^{y-y_{1}-1}$ have the same monotonicity on $[0,1]$.

Applying Chebyshev’s integral inequality (7), for the selected *f*, *g*, and *p*, we have

$$\begin{aligned} & \biggl( \int_{0}^{1} t^{x-1} (1-t)^{y_{1}-1} \exp \biggl( -\frac{ \sigma }{t(1-t)} \biggr)\,dt \biggr) \\ &\qquad {}\times \biggl( \int_{a}^{b} t^{x_{1}-1} (1-t)^{y-1} \exp \biggl( -\frac{ \sigma }{t(1-t)} \biggr)\,dt \biggr) \\ &\quad\leq \biggl( \int_{a}^{b}t^{x_{1}-1} (1-t)^{y_{1}-1} \exp \biggl( -\frac{ \sigma }{t(1-t)} \biggr)\,dt \biggr) \\ &\quad \quad {}\times \biggl( \int_{a}^{b} t^{x-1} (1-t)^{y-1} \exp \biggl( -\frac{ \sigma }{t(1-t)} \biggr)\,dt \biggr), \end{aligned}$$

which is equivalent to (6). □

#### Theorem 2

*The function* $\sigma \mapsto B_{\sigma }(x, y)$ *is log*-*convex on* $(0, \infty )$ *for any fixed* $x, y>0$. *In particular*:

(i) *The functions* $B_{\sigma }(x, y)$ *satisfy the Turán*-*type inequality*
  $$\begin{aligned} B_{\sigma }^{2}(x, y) - B_{\sigma +a}(x, y) B_{\sigma -a}(x, y)\leq 0, \end{aligned}$$
  *for all real* *a*. *This will further reduce to* $B_{\sigma }^{2}(x, y) \leq B(x, y) B_{2\sigma }(x, y)$ *when* $\sigma =a$. *Here* $B(x, y)=B _{0}(x, y)$ *is the classical beta function*.
(ii) *The function* $\sigma \mapsto B_{\sigma }(x-1, y-1)/ B_{ \sigma }(x, y)$ *is decreasing on* $(0, \infty )$ *for any fixed* $x,y >0$.

#### Proof

By the definition of log-convexity it is required to prove that

8 $$\begin{aligned} B_{\alpha \sigma_{1}+(1-\alpha ) \sigma_{2}}(x, y) \leq \bigl( B_{ \sigma_{1}}(x, y) \bigr) ^{\alpha} \bigl( B_{\sigma_{2}}(x, y) \bigr) ^{1-\alpha } \end{aligned}$$

for $\alpha \in [0,1]$, $\sigma_{1}, \sigma_{2} >0$, and fixed $x, y>0$.

Clearly, (8) is trivially true for $\alpha = 0$ and $\alpha =1$.

Let $\alpha \in (0,1)$. It follows from (1) that

9 $$\begin{aligned} B_{\alpha \sigma_{1}+(1-\alpha ) \sigma_{2}}(x, y) ={}& \int_{0} ^{1} t^{x-1} (1-t)^{y-1} \exp \biggl( -\frac{\alpha \sigma_{1}+(1-\alpha )\sigma_{2}}{t(1-t)} \biggr)\,dt \\ =&{} \int_{0}^{1} \biggl( t^{x-1} (1-t)^{y-1} \exp \biggl( -\frac{ \sigma_{1}}{t(1-t)} \biggr)\,dt \biggr) ^{\alpha } \\ &{}\times \int_{0}^{1} \biggl( t^{x-1} (1-t)^{y-1} \exp \biggl( \frac{-\sigma _{2}}{t(1-t)} \biggr)\,dt \biggr) ^{1-\alpha }. \end{aligned}$$

Let $p=1/\alpha $ and $q=1/(1-\alpha )$. Clearly, $p>1$ and $p+q=pq$. Thus, applying the well-known Hölder-Rogers inequality for integrals, (9) yields

10 $$\begin{aligned} B_{\alpha \sigma_{1}+(1-\alpha ) \sigma_{2}}(x, y) < {}& \biggl( \int _{0}^{1} t^{x-1} (1-t)^{y-1} \exp \biggl( -\frac{ \sigma_{1}}{t(1-t)} \biggr)\,dt \biggr) ^{\alpha } \\ & {}\times \biggl( \int_{0}^{1} t^{x-1} (1-t)^{y-1} \exp \biggl( -\frac{\sigma _{2}}{t(1-t)} \biggr)\,dt \biggr) ^{1-\alpha } \\ =&{} \bigl( B_{\sigma_{1}}(x, y) \bigr) ^{\alpha} \bigl( B_{\sigma_{2}}(x, y) \bigr) ^{1-\alpha }. \end{aligned}$$

This implies that $\sigma \mapsto B_{\sigma }(x, y)$ is log-convex.

Choosing $\alpha =1/2$, $\sigma_{1}=\sigma -a$, and $\sigma_{2}= \sigma +a$, inequality (10) gives

$$\begin{aligned} B_{\sigma }^{2}(x, y) - B_{\sigma +a}(x, y) B_{\sigma -a}(x, y)\leq 0. \end{aligned}$$

The log-convexity of $B_{\sigma }(x,y)$ is equivalent to

11 $$\begin{aligned} \frac{\partial }{\partial \sigma } \biggl( \frac{ \frac{\partial }{ \partial \sigma } B_{\sigma }(x, y)}{B_{\sigma }(x, y)} \biggr) \geq 0. \end{aligned}$$

Now the identity [1], p.22,

$$\frac{\partial^{n}}{\partial \sigma^{n}} B_{\sigma }(x, y) = (-1)^{n} B_{\sigma }(x-n, y-n), \quad n=0, 1, 2, \ldots, $$

reduces (11) to

$$\frac{\partial }{\partial \sigma } \biggl( \frac{ B_{\sigma }(x-1, y-1)}{B _{\sigma }(x, y)} \biggr) \leq 0. $$

Hence the conclusion. □

#### Theorem 3

*The function* $(x,y) \mapsto B_{\sigma }( x, y)$ *is logarithmic convex on* $(0, \infty ) \times (0, \infty ) $ *for all* $\sigma \geq 0$. *In particular*,

$$B_{\sigma }^{2} \biggl( \frac{x_{1}+x_{2}}{2}, \frac{y_{1}+y_{2}}{2} \biggr) \leq B_{\sigma } ( x_{1},y_{1} ) B_{\sigma } ( x_{2}, y_{2} ). $$

#### Proof

Let $\alpha_{1}, \alpha_{2} >0$ be such that $\alpha_{1}+\alpha_{2}=1$. Then, for $\sigma \geq 0$, we have

$$\begin{aligned}& B_{\sigma } \bigl(\alpha_{1}( x_{1}, y_{1})+ \alpha_{2}(x_{2},y_{2}) \bigr) \\& \quad = \int_{0}^{1} t^{ \alpha_{1}x_{1}+ \alpha_{2} x_{2}-1} (1-t)^{ \alpha_{1} y_{1}+ \alpha_{2} y_{2}-1} \exp \biggl( - \frac{\sigma }{t(1-t)} \biggr)\,dt \\& \quad = \int_{0}^{1} \biggl( t^{ x_{1}-1} (1-t)^{ y_{1}-1} \exp \biggl( -\frac{ \sigma }{t(1-t)} \biggr) \biggr) ^{\alpha_{1}} \\& \quad \quad {}\times \biggl( t^{ x_{2}-1} (1-t)^{ y_{2}-1} \exp \biggl( - \frac{\sigma }{t(1-t)} \biggr) \biggr) ^{\alpha_{2}}\,dt. \end{aligned}$$

Again by considering $p=1/\alpha_{1}$ and $q=1/\alpha_{2}$, by the Hölder-Rogers inequality for integrals it follows that

$$\begin{aligned} B_{\sigma } \bigl(\alpha_{1}( x_{1}, y_{1})+ \alpha_{2}(x_{2},y_{2}) \bigr) &\leq \biggl( \int_{0}^{1} t^{ x_{1}-1} (1-t)^{ y_{1}-1} \exp \biggl( -\frac{ \sigma }{t(1-t)} \biggr)\,dt \biggr) ^{\alpha_{1}} \\ &\quad \times{} \biggl( \int_{0}^{1} t^{ x_{2}-1} (1-t)^{ y_{2}-1} \exp \biggl( -\frac{\sigma }{t(1-t)} \biggr)\,dt \biggr) ^{\alpha_{2}} \\ &= B_{\sigma }( x_{1}, y_{1})^{\alpha_{1}} B_{\sigma }( x_{2}, y_{2})^{ \alpha_{2}}. \end{aligned}$$

For $\alpha_{1}=\alpha_{2}=1/2$, this inequality reduces to

12 $$\begin{aligned} B_{\sigma }^{2} \biggl( \frac{x_{1}+x_{2}}{2}, \frac{y_{1}+y_{2}}{2} \biggr) \leq B_{\sigma } ( x_{1},y_{1} ) B_{\sigma } ( x_{2}, y_{2} ). \end{aligned}$$

Let $x, y >0 $ be such that

$$\min_{a \in \mathbb{R}}(x+a, x-a) >0. $$

Then $x_{1} = x+a$, $x_{2}=x-a$ and $y_{1}=y+b$, $y_{2}=y-b$ in (12) yields

13 $$\begin{aligned} \bigl[ B_{\sigma }(x, y) \bigr]^{2} \leq B_{\sigma }( x+a, y+b) B_{\sigma} (x-a, y-b) \end{aligned}$$

for all $\sigma \geq 0$. □

The Grüss inequality [14], pp.95-310, for the integrals is given in the following lemma.

#### Lemma 1

*Let* *f* *and* *g* *be two integrable functions on* $[a, b]$. *If*

$$m \leq f(t) \leq M \quad \textit{and} \quad l \leq g(t) \leq L \quad \textit{for each } t \in [a, b], $$

*where* *m*, *M*, *l*, *L* *are given real constants*. *Then*

14 $$\begin{aligned} \bigl\vert D(f, g; h) \bigr\vert \leq D(f, f; h)^{1/2}D(g, g; h)^{1/2} \leq \frac{1}{4} (M-m) (L-l) \biggl[ \int_{a}^{b} h(t)\,dt \biggr] ^{2}, \end{aligned}$$

*where*

$$\begin{aligned} D(f, g; h) := \int_{a}^{b} h(t)\,dt \int_{a}^{b} h(t) f(t) g(t)\,dt- \int_{a}^{b} h(t) f(t)\,dt \int_{a}^{b} h(t) g(t)\,dt. \end{aligned}$$

Our next result is the application of the Grüss inequality for the extended beta mappings.

#### Theorem 4

*Let* $\sigma_{1}, \sigma_{2}, x, y >0$. *Then*

15 $$\begin{aligned} & \bigl\vert B_{\sigma_{1}+\sigma_{2}}(x+y+1, x+y+1) - B_{\sigma _{1}}(x+1, x+1) B_{\sigma_{2}}(y+1, y+1) \bigr\vert \\ &\quad \leq \bigl[ B_{2\sigma_{1}}(2x+1, 2x+1) - B_{\sigma_{1}}(x+1, x+1) ^{2} \bigr] ^{\frac{1}{2}} \\ &\quad \quad {}\times \bigl[ B_{2\sigma _{1}}(2y+1, 2y+1) - B_{\sigma_{1}}(y+1, y+1) ^{2} \bigr] ^{\frac{1}{2}} \\ &\quad \leq \frac{ \exp(-4(\sigma_{1}+\sigma_{2}))}{4^{x+y+1}}. \end{aligned}$$

#### Proof

To prove the inequality, it is required to determine the upper and lower bounds of

$$\begin{aligned} f(t) &: = t^{x} (1-t)^{x} \exp \biggl( -\frac{\sigma_{1}}{t(1-t)} \biggr) \end{aligned}$$

and

$$\begin{aligned} g(t) &: = t^{y} (1-t)^{y} \exp \biggl( -\frac{\sigma_{2}}{t(1-t)} \biggr) \end{aligned}$$

for $t \in [0,1]$ and $x, y, \sigma_{1}, \sigma_{2} >0$. Clearly, $f(0)=f(1)=0$ and $g(0)=g(1)=0$. Now for $t \in (0,1)$, the logarithmic differentiation of *f* yields

$$\begin{aligned} f'(t) = f(t) (1-2t) \biggl( \frac{ x t(1-t)+\sigma_{1}}{t^{2} (1-t)^{2}} \biggr). \end{aligned}$$

Since $f(t)>0 $ and $x t(1-t)+\sigma_{1}>0$ on $t \in (0,1)$, $f'(t) >0$ for $t>1/2$ and $f'(t) <0$ for $t<1/2$. This implies

$$M = \frac{\exp (- 4 \sigma_{1})}{4^{x}}. $$

Similarly, we can show that

$$L= \frac{\exp (- 4 \sigma_{2})}{4^{y}}. $$

Now setting *f*, *g* as before and $h(t)=1$ for all $t \in [0,1]$ in Lemma 1 gives (15). □

#### Remark 1

Consider the functions

$$f(t) = t^{x}, \qquad g(t)= (1-t)^{y} \quad \text{and} \quad h(t) = t ^{x_{1}-1} (1-t)^{y_{1}-1} \exp \biggl( - \frac{\sigma }{t(1-t)} \biggr) $$

for $t \in [0, 1] $, $x, y, x_{1}, y_{1} >0$. Clearly, $M=L=1$ and $m=l=0$. Thus, from Lemma 1 we have the following inequality:

16 $$\begin{aligned} & \bigl\vert B_{\sigma }(x_{1}, y_{1})B_{\sigma }(x+x_{1}, y+y_{1}) - B_{\sigma }(x+x_{1}, y_{1}) B_{\sigma }(x_{1}, y+y_{1}) \bigr\vert \\ &\quad \leq \bigl[ B_{\sigma }(x_{1}, y_{1}) B_{\sigma }(2x+x _{1}, y_{1}) - B^{2}_{\sigma }(x+x_{1}, y_{1}) \bigr] ^{\frac{1}{2}} \\ & \quad \quad{}\times \bigl[ B_{\sigma }(x_{1}, y_{1}) B_{\sigma }(x_{1}, 2y+y_{1}) - B^{2}_{\sigma }(x_{1},y+ y_{1}) \bigr] ^{\frac{1}{2}} \\ &\quad \leq \frac{ B^{2}_{\sigma }(x_{1}, y_{1})}{4}. \end{aligned}$$

Similarly, if *f*, *g*, and *h* defined as

$$f(t): = t^{m}(1-x)^{n}, \qquad g(t):= t^{p}(1-t)^{q} \quad \text{and} \quad h(t) := t^{\alpha -1} (1-t)^{\beta -1} \exp \biggl( - \frac{\sigma }{t(1-t)} \biggr) $$

for $t \in [0,1]$ and $\alpha, \beta, m, n, p, q>0 $, then (see [11]) we have

$$M= \frac{ m^{m} n^{n}}{(m+n)^{m+n}} \quad \text{and} \quad L=\frac{ p ^{p} q^{q}}{(p+q)^{p+q}}; $$

hence, the inequality

17 $$\begin{aligned} & \bigl\vert B_{\sigma }(\alpha, \beta )B_{\sigma }(\alpha +m+p, \beta +n+q) - B_{\sigma }(\alpha +m, \beta +n) B_{\sigma }(\alpha +p, \beta +q) \bigr\vert \\ &\quad \leq \bigl[ B_{\sigma }(\alpha, \beta ) B_{\sigma }( \alpha +2m, \beta +2n) - B^{2}_{\sigma }(\alpha +m, \beta +m) \bigr] ^{\frac{1}{2}} \\ & \quad \quad \times \bigl[ B_{\sigma }(\alpha, \beta ) B_{\sigma }(\alpha +2p, \beta +2q) - B^{2}_{\sigma }(\alpha +p, \beta +q) \bigr] ^{ \frac{1}{2}} \\ &\quad \leq \frac{ B^{2}_{\sigma }(\alpha, \beta )}{4}\cdot\frac{ m ^{m} n^{n}}{(m+n)^{m+n}}\cdot \frac{ p^{p} q^{q}}{(p+q)^{p+q}} \end{aligned}$$

follows from Lemma 1.

#### Remark 2

It is evident from Theorem 1 and inequalities (16) and (17) that the results discussed in [11, 12] for classical beta functions can be replicated for the extended beta functions.

### Inequalities for ECHFs and EGHFs

Along with the integral inequalities mentioned in the previous section, the following result of Biernacki and Krzyż [15] will be used in the sequel.

#### Lemma 2

[15] *Consider the power series* $f(x)=\sum_{n\geq 0} a_{n} x ^{n}$ *and* $g(x)=\sum_{n\geq 0} b_{n} x^{n}$, *where* $a_{n} \in \mathbb{R}$ *and* $b_{n} > 0$ *for all* *n*. *Further*, *suppose that both series converge on* $|x|< r$. *If the sequence* $\{a_{n}/b_{n}\}_{n\geq 0}$ *is increasing* (*or decreasing*), *then the function* $x \mapsto f(x)/g(x)$ *is also increasing* (*or decreasing*) *on* $(0,r)$.

We note that this lemma still holds when both *f* and *g* are even or both are odd functions.

#### Theorem 5

*Let* $b \geq 0$ *and* $d, c >0$. *Then following assertions for ECHFs are true*:

(i) *For* $c \geq d$, *the function* $x \mapsto \Phi_{\sigma }(b ; c; x)/\Phi_{\sigma }(b ; d; x)$ *is increasing on* $(0, \infty )$.
(ii) *For* $c \geq d$, *we have* $d \Phi_{\sigma }(b+1 ; c+1; x) \Phi_{\sigma }(b ; d; x) \geq c \Phi_{\sigma }(b ; c; x) \Phi_{\sigma }(b+1 ; d+1; x)$.
(iii) *The function* $x \mapsto \Phi_{\sigma }(b ; c; x)$ *is log*-*convex on* $\mathbb{R}$.
(iv) *The function* $\sigma \mapsto \Phi_{\sigma }(b ; c; x)$ *is log*-*convex on* $(0, \infty )$ *for fixed* $x>0$.
(v) *Let* $\delta >0$. *Then the function*
  $$b \mapsto \frac{B(b, c) \Phi_{\sigma }(b +\delta ; c; x)}{B(b+\delta , c) \Phi_{\sigma }(b ; c; x)} $$
  *is decreasing on* $(0, \infty )$ *for fixed* $c, x>0$.

#### Proof

From the definition of ECHFs it follows that

18 $$\begin{aligned} \frac{\Phi_{\sigma }(b ; c; x)}{\Phi_{\sigma }(b ; d; x)} = \frac{ \sum_{n=0}^{\infty} \alpha_{n}(c) x^{n} }{ \sum_{n=0}^{\infty} \alpha _{n}(d) x^{n}}, \quad \text{where } \alpha_{n}(t):=\frac{B_{ \sigma }(b+n, t-b)}{B(b, t-b) n!}. \end{aligned}$$

If we denote $f_{n}= \alpha_{n}(c)/ \alpha_{n}(d)$, then

$$\begin{aligned} f_{n}- f_{n+1} & = \frac{\alpha_{n}(c)}{ \alpha_{n}(d)}-\frac{\alpha _{n+1}(c)}{ \alpha_{n+1}(d)} \\ &= \frac{B(b, d-b)}{B(b, c-b)} \biggl( \frac{B_{\sigma }(b+n, c-b)}{B _{\sigma }(b+n, d-b)}-\frac{B_{\sigma }(b+n+1, c-b)}{B_{\sigma }(b+n+1, d-b)} \biggr). \end{aligned}$$

Now set $x:= b+n$, $y:=d-b$, $x_{1}:=b+n+1$, and $y_{1}:=c-b$ in (6). Since $(x-x_{1})(y-y_{1})=c-d \geq 0$, it follows from Theorem 1 that

$$\frac{B_{\sigma }(b+n, c-b)}{B_{\sigma }(b+n, d-b)} \leq \frac{B_{ \sigma }(b+n+1, c-b)}{B_{\sigma }(b+n+1, d-b)}, $$

which is equivalent to say that the sequence $\{f_{n}\}$ is increasing, and by Lemma 2 we can conclude that $x \mapsto \Phi_{ \sigma }(b ; c; x)/\Phi_{\sigma }(b ; d; x)$ is increasing on $(0, \infty )$.

To prove (ii), we need to recall the following identity from [3], p.594:

19 $$\begin{aligned} \frac{d^{n}}{d x^{n}} \Phi_{\sigma }(b ; c; x)= \frac{(b)_{n}}{(c)_{n}} \Phi_{\sigma }(b+n ; c+n; x). \end{aligned}$$

Now the increasing property of $x \mapsto \Phi_{\sigma }(b ; c; x)/ \Phi_{\sigma }(b ; d; x)$ is equivalent to

20 $$\begin{aligned} \frac{d}{dx} \biggl( \frac{\Phi_{\sigma }(b ; c; x)}{\Phi_{\sigma }(b ; d; x)} \biggr) \geq 0. \end{aligned}$$

This, together with (19), implies

$$\begin{aligned}& \Phi '_{\sigma }(b ; c; x) \Phi_{\sigma }(b ; d; x) - \Phi_{\sigma }(b ; c; x) \Phi '_{\sigma }(b ; d; x) \\& \quad = \frac{b}{c} \Phi_{\sigma }(b+1 ; c+1; x) \Phi_{\sigma }(b ; d; x) - \frac{b}{d}\Phi_{\sigma }(b ; c; x) \Phi_{\sigma }(b+1 ; d+1; x) \geq 0. \end{aligned}$$

A simple computation prove the assertion.

The log-convexity of $x \mapsto \Phi_{\sigma }(b ; c; x)$ can be proved by using the integral representation of ECHFs as given in (3) and by applying to the Hölder-Rogers inequality for integrals as follows:

$$\begin{aligned} &\Phi_{\sigma } \bigl(b ; c; \alpha x+ (1-\alpha ) y \bigr) \\ &\quad =\frac{1}{B(b, c-b)} \int_{0}^{1} t^{b-1} (1-t)^{c-b-1} \exp \biggl( \alpha x t+ (1-\alpha ) y t - \frac{\sigma }{t(1-t)} \biggr)\,dt \\ &\quad =\frac{1}{B(b, c-b)} \int_{0}^{1} \biggl[ \biggl( t^{b-1} (1-t)^{c-b-1} \exp \biggl( x t - \frac{\sigma }{t(1-t)} \biggr) \biggr) ^{\alpha} \\ &\quad \quad {}\times \biggl( t^{b-1} (1-t)^{c-b-1} \exp \biggl( y t- \frac{ \sigma }{t(1-t)} \biggr) \biggr) ^{1-\alpha } \biggr]\,dt \\ &\quad \leq \biggl[\frac{1}{B(b, c-b)} \int_{0}^{1} t^{b-1} (1-t)^{c-b-1} \exp \biggl( x t - \frac{\sigma }{t(1-t)} \biggr)\,dt \biggr]^{\alpha} \\ &\quad \quad {}\times \biggl[\frac{1}{B(b, c-b)} \int_{0}^{1} t^{b-1} (1-t)^{c-b-1} \exp \biggl( y t- \frac{\sigma }{t(1-t)} \biggr)\,dt \biggr]^{1-\alpha } \\ &\quad = \bigl( \Phi_{\sigma }(b ; c; x) \bigr) ^{\alpha} \bigl( \Phi_{\sigma }(b ; c; y) \bigr) ^{1-\alpha }, \end{aligned}$$

where $x, y \geq 0$ and $\alpha \in [0,1]$. This proves that $x \mapsto \Phi_{\sigma }(b ; c; x)$ is log-convex for $x\geq 0$. For the case $x <0$, the assertion follows immediately from the identity ([3], p.596)

$$\Phi_{\sigma }(b ; c; -x)= e^{-x} \Phi_{\sigma }(c-b; c; x). $$

It is known that the infinite sum of log-convex functions is also log-convex. Thus, the log-convexity of $\sigma \mapsto \Phi_{\sigma }(b ; c; x)$ is equivalent to showing that $\sigma \mapsto B_{\sigma }(b+n, c-b)$ is log-convex on $(0, \infty )$ and for all nonnegative integers *n*. From Theorem 2 it is clear that $\sigma \mapsto B_{ \sigma }(b+n, c-b)$ is log-convex for $c>b>0$, and hence (iv) is true.

Let $b' \geq b$. Set $p(t):= t^{b'-1} (1-t)^{c-b'-1} \exp ( x t- \frac{ \sigma }{t(1-t)} ) $,

$$f(t):= \biggl( \frac{t}{1-t} \biggr) ^{b-b'} \quad \text{and} \quad g (t):= \biggl( \frac{t}{1-t} \biggr) ^{\delta }. $$

Then using the integral representation (3) of ECHFs, we have

21 $$\begin{aligned}& \frac{B(b, c) \Phi_{\sigma }(b+\delta ; c; x) }{B(b+\delta, c) \Phi_{\sigma }(b ; c; x)} - \frac{B(b', c) \Phi_{\sigma }(b'+\delta ; c; x) }{B(b'+\delta, c) \Phi_{\sigma }(b' ; c; x)} \\& \quad =\frac{\int_{0} ^{1} f(t) g(t) p(t)\,dt}{\int_{0}^{1} f(t) p(t)\,dt } - \frac{\int_{0} ^{1} g(t) p(t)\,dt}{ \int_{0}^{1}p(t)\,dt }. \end{aligned}$$

It is easy to determine that for $b' \geq b$, the function *f* is decreasing, whereas for $\delta \geq 0$, the function *g* is increasing. Since *p* is nonnegative for $t \in [0,1]$, by the reverse Chebyshev integral inequality (7) it follows that

22 $$\begin{aligned} \int_{0}^{1} p(t) f(t)\,dt \int_{0}^{1} p(t) g(t)\,dt \leq \int_{0}^{1} p(t)\,dt \int_{0}^{1} p(t)f(t) g(t)\,dt. \end{aligned}$$

This, together with (21), implies

$$\begin{aligned} \frac{B(b, c) \Phi_{\sigma }(b+\delta ; c; x) }{B(b+\delta, c) \Phi_{\sigma }(b ; c; x)} - \frac{B(b', c) \Phi_{\sigma }(b'+\delta ; c; x) }{B(b'+\delta, c) \Phi_{\sigma }(b' ; c; x)} \geq 0, \end{aligned}$$

which is equivalent to saying that the function

$$b \mapsto \frac{B(b, c) \Phi_{\sigma }(b +\delta ; c; x)}{B(b+\delta , c) \Phi_{\sigma }(b ; c; x)} $$

is decreasing on $(0, \infty )$. □

#### Remark 3

In particular, the decreasing property of

$$b \mapsto \frac{B(b, c) \Phi_{\sigma }(b +\delta ; c; x)}{B(b+\delta , c) \Phi_{\sigma }(b ; c; x)} $$

is equivalent to the inequality

23 $$\begin{aligned} \Phi_{\sigma }^{2}(b+\delta ; c; x) \geq \frac{ B^{2}(b+\delta, c)}{ B(b+2 \delta, c) B(b, c)} \Phi_{\sigma }(b+2\delta ; c; x) \Phi_{ \sigma }(b; c; x). \end{aligned}$$

Now define

$$f(\delta ) := \frac{ B^{2}(b+\delta, c)}{ B(b+2 \delta, c) B(b, c)}= \frac{ (\Gamma (b + \delta ))^{2} \Gamma (b + 2 \delta +c) \Gamma (b+c)}{( \Gamma ( b+c + \delta ))^{2} \Gamma (b+ 2 \delta ) \Gamma (b)}. $$

A logarithmic differentiation of *f* yields

$$\begin{aligned} \frac{f'(\delta )}{f(\delta )} = 2 \psi (b + \delta ) + 2 \psi (b + 2 \delta + c)- 2 \psi (b+c + \delta ) - 2 \psi (b + 2 \delta ), \end{aligned}$$

where $y \mapsto \psi (y)=\Gamma '(y)/\Gamma (y)$ is the digamma function, which is increasing on $(0,\infty )$ and has the series form

$$\psi (y)=-\gamma + \sum_{k\geq 0} \biggl( \frac{1}{k}-\frac{1}{y+k} \biggr). $$

This implies that

$$\begin{aligned} \frac{f'(\delta )}{f(\delta )} &= 2 \sum_{k=0}^{\infty} \biggl( \frac{1}{b+c+ \delta +k}-\frac{1}{b+c+2\delta +k} \biggr) + 2 \sum _{k=0}^{\infty} \biggl( \frac{1}{b+\delta +k}- \frac{1}{b+2\delta +k} \biggr) \\ &= 2 \delta \sum_{k=0}^{\infty} \biggl( \frac{1}{(b+c+\delta +k)(b+c+2 \delta +k) }- \frac{1}{(b+\delta +k)(b+2\delta +k)} \biggr) \\ &= - 2 \delta \sum_{k=0}^{\infty} \frac{c(2b+3 \delta + 2 k+c)}{(b+c+ \delta +k)(b+c+2\delta +k) (b+\delta +k)(b+2\delta +k)} \leq 0. \end{aligned}$$

Thus, *f* is a decreasing function of *δ* on $[0, \infty )$, and $f(\delta ) \leq f(0)=1$.

Interestingly, for $\sigma =0$, inequality (23) reduces to the Turán-type inequality of classical confluent hypergeometric functions

24 $$\begin{aligned} {}_{1}F_{1}^{2}(b+\delta ; c; x) \geq \frac{ B^{2}(b+\delta, c)}{ B(b+2 \delta, c) B(b, c)} {}_{1}F_{1}(b+2\delta ; c; x) {}_{1}F_{1}(b; c; x). \end{aligned}$$

Since

$$\frac{ B^{2}(b+\delta, c)}{ B(b+2 \delta, c) B(b, c)} \leq 1, $$

we can conclude that inequality (24) is an improvement of the inequality given in [9], Theorem 4(b), for fixed $c, x>0$. However, our result does not expound the other cases in [9], Theorem 4(b).

Now following the remark given in [9], p.390, for integer *δ* and $b=\delta +a$ in (24), will also improve inequality ([10], Theorem 1, Corollary 2), for classical confluent hypergeometric functions.

Our next result is on the extended Gaussian hypergeometric functions (EGHFs).

#### Theorem 6

*Let* $b \geq 0$ *and* $d, c >0$. *Then following assertions for EGHFs are true*.

(i) *For* $c \geq d$, *the function* $x \mapsto F_{\sigma }(a, b ; c; x)/F_{\sigma }(a, b ; d; x)$ *is increasing on* $(0, 1)$.
(ii) *For* $c \geq d$, *we have*
  $$d F_{\sigma }(a+1, b+1 ; c+1; x) F_{\sigma }(a, b ; d; x) \geq c F _{\sigma }(a+1, b+1 ; d+1; x) F_{\sigma }(a, b ; c; x). $$
(iii) *The function* $\sigma \mapsto F_{\sigma }(a, b ; c; x)$ *is log*-*convex on* $(0, \infty )$ *for fixed* $b>0, c>0$, *and* $x \in (0, 1)$.
(iv) *The function* $a \mapsto F_{\sigma }(a, b ; c; x)$ *is log*-*convex on* $(0, \infty )$ *and for fixed* $x \in (0, 1)$.

#### Proof

Cases (i)-(iii) can be proved by following the proof of Theorem 5 and considering the series form (4) and an integral representation (5) of EGHFs, we omit the details.

From a result of Karp and Sitnik [9] we know that if

$$f(a,x)=\sum_{n\geq 0}f_{n} \frac{(a)_{n}}{n!} x^{n}, $$

where $f_{n}$ is independent of *a*, and we suppose that $a'>a>0$ and $\delta >0$, then the function

$$f(a+\delta,x)f(b,x)-f(b+\delta,x)f(a,x)=\sum_{m\geq 2} \phi_{m} x ^{m} $$

has negative power series coefficient $\phi_{m} < 0$, so that $a \mapsto f(a,x)$ is strictly log-convex for $x>0$ if the sequence $\{ f_{n}/f_{n-1} \} $ is increasing. In what follows, we use this result for the function $F_{\sigma }(a, b; c; x)$. For this, let

$$f_{n}=\frac{ B_{\sigma }(b+n, c-b)}{B(b, c-b)}. $$

Thus, to prove (iv), it suffices to show that the sequence $d_{n} = f _{n}/ f_{n-1}$ is decreasing. Clearly,

$$d_{n}- d_{n-1} = \frac{B_{\sigma }(b+n, c-b)}{B_{\sigma }(b+n-1, c-b)}- \frac{B_{ \sigma }(b+n-1, c-b)}{B_{\sigma }(b+n-2, c-b)}. $$

Now if we replace $x_{1}, y_{1}, x_{2}, y_{2}$ in (12) by $x_{1}=b+n$, $x_{2}=b+n-2$, and $y_{1}=y_{2}=c-b$, then it follows that $d_{n} \geq d_{n-1}$. Hence the conclusion. □

## Conclusion

In this article, we prove several properties of the extended beta functions resembling the classical beta functions. A few of those properties are a key to establish inequalities for ECHFs and EGHFs. Using classical integral inequalities, we also give Turán-type and reverse Turán-type inequalities for ECHFs and EGHFs.
